# A Comparison of Symptom Improvement and Outcomes After Septoplasty Alone Versus Septoplasty With Turbinoplasty

**DOI:** 10.7759/cureus.36628

**Published:** 2023-03-24

**Authors:** Khalid T Hakami, Zohour A Almalki, Farah S Alnemari, Raneem M Alotaibi, Fatimah R Bajunaid

**Affiliations:** 1 Department of Otolaryngology, Head and Neck Surgery, Rhinology Unit, Al-Hada Armed Forces Hospital, Taif, SAU; 2 Department of Medicine and Surgery, Taif University, Taif, SAU

**Keywords:** postoperative complication, turbinates, nasal septum, nasal surgical procedures, nasal obstruction

## Abstract

Background

Various causes can lead to nasal obstruction, with the most frequent anatomical cause being deviated nasal septum. It seriously affects patients’ quality of life. As a result, septoplasty is performed to enhance the nasal airways. This study aimed to compare the improvement of nasal symptoms following septoplasty with or without turbinoplasty and evaluate the surgical outcomes in both different groups.

Methodology

A retrospective study was conducted at a tertiary hospital among patients who had undergone septoplasty with or without turbinoplasty between 2020 and 2022. Data regarding demographics, clinical features, surgical data, and complications were collected from patient files. The Nasal Obstruction Symptom Evaluation (NOSE) scale score was assessed through structured interviews.

Results

In our analysis of 209 patients who underwent surgery for deviated nasal septum, septoplasty was done in 110 (52.6%) patients, whereas the remaining 99 (47.4%) underwent septoplasty with turbinoplasty. The mean NOSE score was found to be 32.94 ± 35.67%. Patients who underwent septoplasty alone significantly showed higher mean scores (56.36 ± 34.62%) compared to those who underwent septoplasty with turbinoplasty (11.14 ± 18.93%) (p < 0.001). The long-term complications showed revision surgery was done in 13 patients, which was comparatively more often done in patients who underwent a septoplasty. Other long-term complications were found to be significantly higher in patients who underwent septoplasty (76.9%) compared to those who underwent septoplasty with turbinoplasty (23.1%).

Conclusions

Patients who underwent additional turbinoplasty experienced an improvement in nasal symptoms than those who underwent septoplasty alone. In addition, more long-term complications were noted in patients who underwent septoplasty alone.

## Introduction

Nasal obstruction is a common symptom in otolaryngology outpatient clinics [1]. It affects up to one-third of the population and prompts individuals to seek medical help [2]. Recently, nasal obstruction has become more widely recognized because it seriously affects the quality of life and various dimensions of daily and social activities [3]. Although there are various causes of nasal obstruction, the most frequent anatomical cause is the deviated nasal septum [4]. Approximately 80% of individuals have a nasal septum that is not precisely on the midline; however, only a few have severe deviation that causes nasal obstruction [5]. In septal deviation to one nostril, compensatory inferior turbinate hypertrophy is thought to protect the airways from extra air entering via that nostril and its potential negative effects [6]. As a result, septoplasty, which is one of the most common procedures performed in the otorhinolaryngology department, is performed to enhance the nasal airways [7,8]. The most common complication of septoplasty is excessive bleeding, but other complications, such as septal hematomas, perforations, infections, nasal blockage, intranasal adhesions, and numbness or sensitivity of the upper teeth or lip, may occur [8]. Many studies have reported that after septoplasty, compensatory hypertrophy naturally regresses without surgical intervention [9]. However, others have asserted that turbinate hypertrophy involves both bone and mucosa, and it has been proposed that these alterations should be treated by performing both nasal septum surgery and turbinoplasty, which reduces the size of the inferior turbinates [8-10]. Furthermore, there is no defined method for determining the type of surgery required; therefore, many otorhinolaryngologists perform septoplasty with or without turbinoplasty, with surgical methods mainly relying on clinical judgment [11]. Previous research showed that septoplasty with and without turbinoplasty successfully relieved nasal obstruction compared to baseline measurements. However, the symptomatic improvement was more pronounced in patients who underwent the combined procedure than in patients who underwent septoplasty alone [12]. Some studies have suggested that septoplasty with turbinoplasty reduces the chance of revision nasal surgery [13]. Our study aimed to assess the outcomes of septoplasty with or without turbinoplasty and the associated short-term and long-term complications; furthermore, it compared the improvement in nasal symptoms after surgery in both patient groups.

## Materials and methods

Study design

This retrospective, non-randomized, observational study was conducted in the Department of Otolaryngology, Head, and Neck Surgery of Al-Hada Armed Forces Hospital in Taif, Saudi Arabia. It included patients who had undergone septoplasty with or without turbinoplasty between 2020 and 2022.

Ethical considerations

The study proposal was reviewed and approved by the research ethics committee of Al-Hada Armed Forces Hospital, Taif, Saudi Arabia (application number: 2022-610). The list of patients was provided by the Department of Otolaryngology, Head, and Neck Surgery. The data were collected by the research team.

Study population

This study included 209 patients who were admitted for septoplasty with or without turbinoplasty between 2020 and 2022.

Inclusion and exclusion criteria

We included both male and female patients older than 16 years of age. However, we excluded patients younger than 16 years, those with nasosinusal tumors and polyps, those with adenoid and hyperplastic pharyngeal tonsils and other nasopharyngeal masses, those with a history of functional endoscopic sinus surgery and polypectomy, and those with craniofacial malformation.

Data collection

Data were collected from the medical files of the patients. Structured interviews were conducted, and the results were entered into an Excel data sheet. Two main categories of data were collected. The first category included sociodemographic data, presenting symptoms, time of diagnosis, date of surgery, associated complications, degree of deviated nasal septum, degree of inferior turbinate hypertrophy, and relevant postoperative and follow-up data. The second category included the Nasal Obstruction Symptom Evaluation (NOSE) scale score, which was assessed by interviewing the patients. The NOSE questionnaire is a well-established scale used for the subjective assessment of nasal obstruction. It included five questions about the following: nasal congestion or stuffiness, nasal blockage or obstruction, trouble breathing through the nose, trouble sleeping, and obstruction during exertion. It evaluates five different symptoms of nasal obstruction which are rated using a five-point Likert scale (0-4) depending on the severity of symptoms (0 = minimum severity, and 4 = maximum severity). The total score ranges between 0 and 20; additionally, the total score is divided by 20 and multiplied by 100 to obtain the score percentage. A higher score indicates more severe nasal obstruction. The validated NOSE scale translated to Arabic was used to evaluate improvement in the airway postoperatively [14].

Statistical analysis

All statistical analyses were performed using SPSS version 23 software (IBM Corp., Armonk, NY USA). Continuous variables were expressed as means and standard deviations and analyzed using the t-test. Categorical variables were expressed as percentages and analyzed using Pearson’s chi-square test. Student’s t-test and analysis of variance were applied to see any statistically significant differences in mean NOSE scores between categorical variables. A multivariate analysis was performed to assess the linear correlation between independent variables (patient factors) with NOSE scores. Statistical significance was set at p-values <0.05.

## Results

Our analysis included 209 patients who underwent surgery for a deviated nasal septum at a tertiary care hospital. The sociodemographic analysis showed that 92 (44%) patients were 18-25 years of age, 152 (72.7%) patients were male, 207 (99%) patients were Saudi citizens, 189 (90.4%) patients resided in Taif, and 145 (69.4%) patients were married. The body mass index of the patients was assessed, and it was found that 81 (38.8%) patients were overweight and only one (0.5%) was obese. Approximately 14 (6.7%) patients had asthma, and 29 (13.9%) patients were tobacco smokers (Table 1).

**Table 1:** Sociodemographics characteristics of the patients.

		N	%
Age (years)	<18	15	7.2
	18–25	92	44.0
	26–35	58	27.8
	36–45	35	16.7
	46–55	7	3.3
	>55	2	1.0
Gender	Female	57	27.3
	Male	152	72.7
Nationality	Saudi	207	99.0
	Non-Saudi	2	1.0
Residence	Inside Taif	189	90.4
	Outside Taif	20	9.6
Marital status	Single	145	69.4
	Married	64	30.6
Body mass index	Underweight	25	12.0
	Normal	93	44.5
	Overweight	81	38.8
	Obese	1	0.5
	Not recorded‎/Not available	9	4.3
Comorbidities and smoking	Diabetes mellitus	3	1.4
	Hypertension	2	1.0
	Asthma	14	6.7
	Hypothyroidism	4	1.9
	Others	10	4.7
	Smoking	29	13.9

Septoplasty was performed for 110 (52.6%) patients; however, the remaining patients (47.4%) underwent septoplasty with turbinoplasty. The presenting symptoms indicated bilateral nasal obstruction in 104 (45%) patients; however, no statistically significant association with the type of surgery was observed (p = 0.994). The duration of nasal obstruction was recorded for only 24 patients, and no statistically significant association with the type of surgery performed was observed (p = 0.265). Twenty-five (12%) patients had nasal discharge; of those patients, 17 underwent septoplasty with turbinoplasty and eight underwent septoplasty alone, showing a statistically significant association (p = 0.013). No statistically significant association was observed between the surgery type and other symptoms such as loss of smell, facial pain, headache, postnasal drip, and epistaxis (p > 0.05). Approximately 27 patients experienced snoring; among those patients, 18 (66.6%) underwent septoplasty with turbinoplasty, showing a statistically significant association (p = 0.028). Features such as external nasal deformity, side of the deviated nasal septum, and degree of deviated nasal septum did not exhibit any statistically significant association with the type of surgery performed (p > 0.05). Furthermore, 120 (57.4%) patients had bilateral inferior turbinate hypertrophy; among those patients, 72 (60%) underwent septoplasty with turbinoplasty (p < 0.001). There was also a statistically significant association between the grade of inferior turbinate hypertrophy and the type of surgery performed. Patients with higher grades (grade ≥3) underwent septoplasty with turbinoplasty on both sides (p < 0.001).

**Table 2:** Comparison of presenting symptoms and characteristics related to deviated nasal septum and inferior turbinate hypertrophy based on the type of surgery.

		Surgery type				P-value
		Septoplasty		Septoplasty + turbinoplasty		
		N	%	N	%	
Nasal obstruction	No obstruction	4	3.6	3	3.0	0.994
	Left side	29	26.4	24	24.2	
	Right side	27	24.5	26	26.3	
	Bilateral	49	44.5	45	45.5	
	NA	1	0.9	1	1.0	
Duration of nasal obstruction	1–2 years	1	0.9	0	0	0.265
	3–5 years	4	3.6	4	4.0	
	6–9 years	1	0.9	0	0	
	≥10 years	4	3.6	10	10.1	
	NA	100	90.9	85	85.9	
Nasal discharge	No	102	92.7	79	79.8	0.013
	Yes	8	7.3	17	17.2	
	NA	0	0	3	3.0	
Loss of smell	No	102	92.7	86	86.9	0.195
	Yes	8	7.3	11	11.1	
	NA	0	0	2	2.0	
Facial pain	No	103	93.6	90	90.9	0.316
	Yes	7	6.4	7	7.1	
	NA	0	0	2	2.0	
Headache	No	96	87.3	81	81.8	0.242
	Yes	14	12.7	16	16.2	
	NA	0	0	2	2.0	
Postnasal drip	No	106	96.4	92	92.9	0.282
	Yes	4	3.6	5	5.1	
	NA	0	0	2	2.0	
Snoring	No	101	91.8	79	79.8	0.028
	Yes	9	8.2	18	18.2	
	NA	0	0	2	2.0	
Epistaxis	No	106	96.4	90	90.9	0.169
	Yes	4	3.6	7	7.1	
	NA	0	0	2	2.0	
External nasal deformity	No	98	89.1	93	93.9	0.212
	Yes	12	10.9	6	6.1	
Side of deviated nasal septum	Right	52	47.3	39	39.4	0.118
	Left	43	39.1	52	52.5	
	Not available	15	13.6	8	8.1	
Degree of deviated nasal septum	Mild	4	3.6	4	4.0	0.479
	Moderate	20	18.2	20	20.2	
	Severe	30	27.3	35	35.4	
	NA	56	50.9	40	40.4	
Inferior turbinate hypertrophy	Right	18	16.4	14	14.1	<0.001
	Left	25	22.7	8	8.1	
	Bilateral	48	43.6	72	72.7	
	NA	19	17.3	5	5.1	
Grades of Right ITH	0	26	23.6	8	8.1	<0.001
	1	0	0	2	2.0	
	2	22	20.0	14	14.1	
	3	12	10.9	40	40.4	
	NA	50	45.5	35	35.4	
Grades of Left ITH	0	20	18.2	14	14.1	<0.001
	1	1	0.9	2	2.0	
	2	20	18.2	14	14.1	
	3	13	11.8	35	35.4	
	NA	56	50.9	34	34.3	

**Figure 1:**
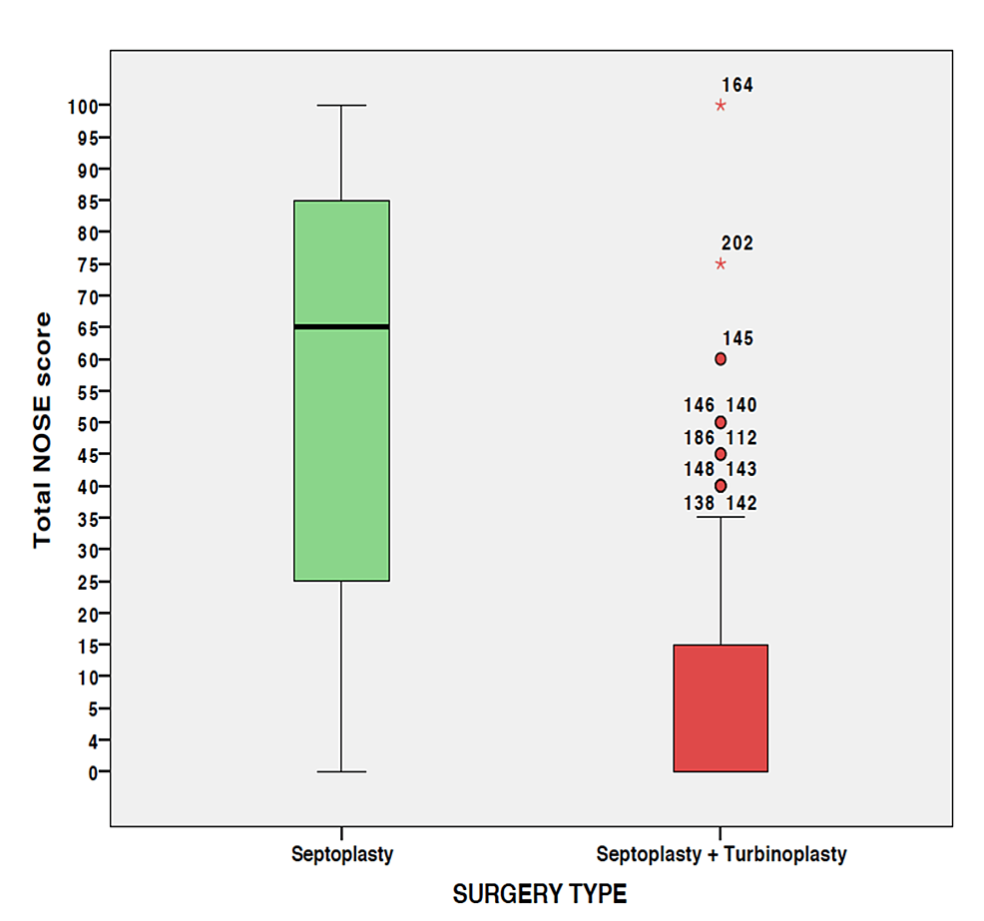
Comparison of NOSE scores (percentage) after the two surgery types. NOSE: Nasal Obstruction Symptom Evaluation

The NOSE scores of 168 patients were recorded. The mean NOSE score was 32.94% (±35.67%). When these scores for the two types of surgery were compared, it was observed that patients who underwent septoplasty alone had significantly higher mean scores (56.36% ± 34.62%) than those who underwent septoplasty with turbinoplasty (11.14% ± 18.93%) (p < 0.001) (Figure 1).

Short-term and long-term complications were assessed after surgery. Few short-term complications were reported. Only one patient developed septal perforation. More long-term complications were reported. Bleeding and infections were reported by one patient, and septal perforation was reported by two patients. Revision surgery was performed for 13 patients. Comparatively, revision surgery was more common among patients who underwent septoplasty (76.9%); however, this result was not statistically significant (p = 0.184). Approximately 13 patients had other long-term complications. The long-term complication rate was significantly higher among patients who underwent septoplasty alone (76.9%) compared to patients who underwent septoplasty with turbinoplasty (23.1%; p < 0.001) (Table 3).

**Table 3:** Comparison of complications based on the type of surgery.

Complications			Surgery type				
			Septoplasty		Septoplasty + turbinoplasty		P-value
			N	%	N	%	
Short-term complications	Bleeding	No	110	100.0	99	100.0	NC
	Infections	No	110	100.0	99	100.0	NC
	Septal perforation	No	103	93.6	94	94.9	0.629
		Yes	1	0.9	0	0	
		NA	6	5.5	5	5.1	
	Others	No	100	90.9	90	90.9	0.981
		Yes	4	3.6	4	4.0	
		NA	6	5.5	5	5.1	
Long-term complications	Bleeding	No	97	88.2	88	88.9	0.636
		Yes	1	0.9	0	0	
		NA	12	10.9	11	11.1	
	Infections	No	97	88.2	88	88.9	0.636
		Yes	1	0.9	0	0	
		NA	12	10.9	11	11.1	
	Septal perforation	No	97	88.2	87	87.9	0.996
		Yes	1	0.9	1	1.0	
		NA	12	10.9	11	11.1	
	Revision surgery	No	90	81.8	85	85.9	0.184
		Yes	10	9.1	3	3.0	
		NA	10	9.1	11	11.1	
	Other	No	67	60.9	81	81.8	<0.001
		Yes	33	30.0	7	7.1	
		NA	10	9.1	11	11.1	

We performed an analysis of covariance to determine the main and interaction effects of the surgery type on NOSE scores by controlling for other independent variables. The analysis showed that there were statistically significant differences in NOSE scores. Patients who underwent septoplasty alone had higher scores than those who underwent septoplasty with turbinoplasty [F (1, 206) = 8.64; p = 0.004] (Table 4).

**Table 4:** Multivariate analysis of NOSE scores. ^a^: R^2^ = 0.073 (adjusted R^2^ = 0.026). BMI: body mass index; NOSE: Nasal Obstruction Symptom Evaluation

Dependent variable: Total NOSE scores					
Source	Type III sum of squares	df	Mean square	F	P-value
Corrected model	20,052.701^a^	10	2005.270	1.545	0.126
Intercept	0.000	0	.	.	.
Hypertension	921.220	1	921.220	0.710	0.401
Smoking	80.079	1	80.079	0.062	0.804
Asthma	12.041	1	12.041	0.009	0.923
Allergic rhinitis	0.000	0	.	.	.
BMI >25 (kg/m^2^)	482.344	1	482.344	0.372	0.543
Bleeding (short-term complications)	0.000	0	.	.	.
Infections (short-term complications)	0.000	0	.	.	.
Septal perforation (short-term complications)	0.740	1	0.740	0.001	0.981
Bleeding (long-term complications)	77.614	1	77.614	0.060	0.807
Infections (long-term complications)	25.274	1	25.274	0.019	0.889
Septal perforation (long-term complications)	356.747	1	356.747	0.275	0.601
Revision surgery	244.954	1	244.954	0.189	0.664
Surgery = septoplasty alone	17,876.148	1	17,876.148	13.773	0.000
Error	253,096.993	195	1,297.933		
Total	388,233.000	206			
Corrected total	273,149.694	205			

## Discussion

Both septoplasty and turbinoplasty can be performed for patients with nasal congestion. For some severe cases, ear, nose, and throat (ENT) surgeons choose turbinoplasty with septoplasty [11,12]. ENT surgeons may adopt different approaches and surgical devices for septoplasty and turbinoplasty. The lack of uniformity in diagnostic procedures and patient decisions complicates the cases and allows surgeons to interpret symptoms and clinical outcomes differently. The overall NOSE symptoms improved after the first 12 months of follow-up in septoplasty combined with turbinoplasty than in septoplasty alone in this study of 209 patients with structural nasal obstruction diagnosed and operated on at a single tertiary hospital. Our research findings are supported by the majority of previous studies. A prospective study by Gandomi et al. assessed postoperative symptoms of 86 young adults with septal deviation at three months and six months after surgery using the NOSE scale and reported that patients who underwent septoplasty with turbinoplasty experienced a statistically significant improvement in symptoms compared to those who underwent septoplasty alone [15]. Another prospective study by Devseren et al. [16] evaluated the six-month postoperative NOSE scores of two groups of patients who underwent septoplasty with and without submucosal resection of the contralateral hypertrophied turbinate and found that subjective symptoms had improved more in the combined intervention group at six months after surgery, although the intranasal NOSE scores decreased in both groups. For the majority of deviated nasal septum and inferior turbinate hypertrophy cases, the average volume of the inferior turbinate is greater; however, performing turbinoplasty can reduce this larger volume, thereby increasing airflow and improving symptoms [17]. A meta-analysis by Leong et al. [18] provided additional evidence that septoplasty with turbinoplasty improved symptoms.

The establishment of a classification for septal deviation has been attempted repeatedly to more effectively compare and standardize the surgical techniques. In particular, it is important to highlight the classifications proposed by Guyuron et al. [19] and Sciuto et al. [20]. Both classifications use the distinction between horizontal, vertical, and combined deviations. Modification of the surgical method is recommended if the septal deviation can be clearly classified. The success rates for this diagnostic and therapeutic procedure have significantly improved, but it is practically impossible to determine whether this improvement is attributable to the classification method or the greater experience of surgeons. Because of the difficulty in isolating a single causal factor, the final decision regarding the appropriate surgical approach relies heavily on the level of expertise and experience of the surgeon. Surgery should be planned after considering the symptoms, any obvious nasal diseases, and, if possible, the results of additional diagnostic tests.

Many of the reported complications following septoplasty are attributable to errors in either the indication or the technical execution of the surgery. However, there is a lack of reliable research on the potential complications associated with septoplasty. In our follow-up, the short-term complications after both types of procedures were very few. However, long-term complications were comparatively more in patients who underwent septoplasty alone than in combination with turbinoplasty. Postsurgical bleeding occurs in 2%-7% of septoplasty patients [21]. Bleeding after turbinoplasty is common, and turbinate resections require nasal packing. Infections are also potential complications. For most cases, the infection rate is less than 3% despite the presence of potentially dangerous bacteria such as staphylococci or streptococci [22-25]. An increased rate of complications is observed with revision surgeries and more complex surgeries. Pirsig and Schäfer reported that 75% of revision surgeries resulted in a mild infection rate of 25% and a serious infection rate of 6% [26]. Based on these findings, it is often suggested that septoplasty should be performed without antibiotic treatment. Although the data from research studies are insufficient, the majority of researchers advocate the use of antibiotics for revision surgeries and complications such as hematomas [27]. Other complications that could occur after septoplasty include septum perforation (3%-25%), hematomas (2%-7%), abscesses, intranasal adhesions (synechia), changes in the outer form of the nose, smelling disorders, and rare complications such as dural injury of the anterior skull base with cerebrospinal leakage and impaired visual function [28-30].

This study had some limitations. First, the outcomes of our analysis were mostly based on subjective measures, such as the NOSE scale. However, objective tools, such as rhinomanometry and nasal flow meters, cannot be used to compare surgical results. Second, the surgeries were not performed by a single surgeon. This could have caused differences in outcomes attributable to variations in surgical execution techniques and the skills and experience of the surgeons.

## Conclusions

We found that patients who underwent turbinoplasty in addition to septoplasty experienced an improvement in nasal symptoms compared with those who underwent septoplasty alone. Long-term complication rates were higher among patients who underwent septoplasty alone than among those who underwent septoplasty with turbinoplasty.
